# How to Give Iron

**Published:** 1888-06

**Authors:** 


**How to Give Iron.**—Most people eat food containing tannic acid; tanic acid unites with iron to form an insoluble tannate of iron. One hour after eating, the tannic acid will have passed from the stomach. Then the iron should be given, and those who have heretofore been disappointed in the use of iron will be surprised at the beneficial results obtainable from the agent.—*Kansas City Medical Index.*

**Bobby**—Ma, did the doctor bring me in the day time or night-time? Mother—in the nighttime, Bobby. Bobby—Well, I guess that's the reason I don't remember anything about it. I must have been asleep.—*Exchange.*

**Sciatica.**—It is stated (*Lancet*) that enveloping the limb for one night in flowers of sulphur, will cure sciatica. The urine next morning smelled strongly of sulphuretted hydrogen.—*Texas Courier-Record.*

